# Evaluation of the introduction of QOF quality improvement modules in English general practice: early findings from a rapid, qualitative exploration of implementation

**DOI:** 10.1136/bmjoq-2022-001960

**Published:** 2022-09-26

**Authors:** Donna Bramwell, Sarah Hotham, Stephen Peckham, Kath Checkland, Lindsay J L Forbes

**Affiliations:** 1 Centre for Primary Care and Health Services Research, Health Organisation, Policy and Economics (HOPE) Group, University of Manchester, Manchester, UK; 2 Centre for Health Services Studies, University of Kent, Canterbury, UK; 3 Department of Health Services and Policy Research, London School of Hygiene and Tropical Medicine, London, UK

**Keywords:** Quality improvement, general practice, PRIMARY CARE, Qualitative research

## Abstract

**Background:**

A 2018 review of the English primary care pay-for-performance scheme, the Quality and Outcomes Framework, suggested that it should evolve to better support holistic, patient-centred care and leadership for quality improvement (QI). From 2019, as part of the vision of change, financially incentivised QI cycles (initially in prescribing safety and end-of-life care), were introduced into the scheme.

**Objectives:**

To conduct a rapid evaluation of general practice staff attitudes, experiences and plans in relation to the implementation of the first two QI modules. This study was commissioned by NHS England and will inform development of the QI programme.

**Methods:**

Semistructured telephone interviews were conducted with 25 practice managers from a range of practices across England. Interviews were audio recorded with consent and transcribed verbatim. Anonymised data were reflexively thematically analysed using the framework method of analysis to identify common themes across the interviews.

**Results:**

Participants reported broadly favourable views of incentivised QI, suggesting the prescribing safety module was easier to implement than the end-of-life module. Additional staff time needed and challenges of reviewing activities with other practices were reported as concerns. Some highlighted that local flexibility and influence on subject matter may improve the effectiveness of QI. Several questioned the choices of topic, recognising greater need and potential for improving quality of care in other clinical areas.

**Conclusion:**

Practices supported the idea of financial incentivisation of QI, however, it will be important to ensure that focus on QI cycles in specific clinical areas does not have unintended effects. A key issue will be keeping up momentum with the introduction of new modules each year which are time consuming to carry out for time poor General Practitioners (GPs)/practices.

**WHAT IS ALREADY KNOWN ON THIS TOPIC**

- Incentivising quality improvement rather than achievement of quality indicators is novel in general practice. Little is known about how this would be received in general practice and what might make it work better.

**WHAT THIS STUDY ADDS**

- Practices highlighted a need for local flexibility and importance of guarding against unintended effects resulting from deprioritisation of other activities.

**HOW THIS STUDY MIGHT AFFECT RESEARCH, PRACTICE OR POLICY**

- Our findings will feed into development of further modules by NHS England, and thus will impact policy and practice.

## Introduction

In the UK, pay-for-performance was introduced into the national contract for general medical practitioners in 2004 in the form of the Quality and Outcomes Framework (QOF). The QOF provides financial reward to practices (not to individual doctors) based on performance on defined indicators. Over its first few years, QOF reduced geographical variation in general practice quality (at least as measured by QOF indicator achievement), led to near universal adoption of electronic medical records and promoted multidisciplinary team-working for long-term conditions.^1^ Nearly all practices now take part, investing considerable effort into maximising achievement; QOF generates, on average, 8% of practice income.^2^

QOF has, however, attracted criticism for its unintended consequences. The standards are largely static—once achieved there is no financial incentive to do any better. While they are supported by robust clinical evidence,^3^ the standards have a narrow focus on processes for single diseases and biological outcomes such as cholesterol levels or blood pressure, and not necessarily what is important to patients.^4^ Related to this, the QOF is seen to limit the exercise of professional judgement and autonomy and is therefore a barrier to patient-centred, holistic management of patients as individuals.^4–7^ Moreover, incentivising only some activities and outcomes—those that are more easily measurable—at the expense of others (eg, continuity of care, quality of the health professional–patient relationship or patient health, well-being or empowerment), may mean that, overall, QOF obstructs rather than promotes achieving high-quality care.^8^

A review of the QOF in 2018, set out a vision for change to promote holistic care and promote quality improvement (QI), rather than focusing solely on attainment of static standards.^4^ Operationalisation of QI for the purposes of UK primary healthcare has been led and articulated by the Royal College of General Practitioners (RCGP), in collaboration with other organisations, and has been widely adopted in the UK.^9 10^ This model involves: continuous organisational commitment to improving outcomes; a set of values, including reflection, teamwork, learning and leadership; and what is called the QI cycle: a structured approach to identify and analyse areas for improvement, plan activities to address them, measure their effects and discuss and share the findings to generate change, iteratively. Most general practices in the UK report that they carry out QI activities,^11^ although the extent to which these follow the RCGP definition of QI is unclear. Furthermore, incentivising QI activities rather than achievement of quality indicators is novel in general practice and to date, little is known about how this would be received in general practice and what might make it work better.

From April 2019, the QOF incentivised QI for the first time, financially rewarding practices for undertaking QI cycles using the RCGP model in two areas, called modules: prescribing safety and end-of-life care, with plans to change the topics on an annual basis.^12^ Table 1 summarises what practices were required to do to receive payment in 2019–2020. As well as conducting QI cycles, practices were required to discuss their activities for the purposes of reflective learning with other practices in their primary care networks (PCNs)—these are collaborative groups of practices serving 30 000–50 000 patients, set up in 2019.^13^

**Table 1:** QI modules 2019/2020 – activity requirements

**Prescribing safety** 37 points available (27 points for activity 10 points for network activity)	Demonstrate continuous QI activity focused on prescribing safety as specified in the QOF guidance and lead to improvements in the following aspects: Reduce the rate of potentially hazardous prescribing, focus on the safer use of non-steroidal anti-inflamatory drugs (NSAIDs) in patients at significant risk of complications such as gastrointestinal bleeding. Better monitoring of potentially toxic medications and the creation of safe systems to support drug monitoring through a focus on lithium prescribing (or another agreed medication if no patients on the registered list are currently being prescribed lithium). Better engagement of patients with their medication through a focus on valproate and pregnancy prevention. Improve collaboration between practices, networks and community pharmacists to share learning and improve systems to reduce harm and improve safety. Participate in a minimum of 2 network peer review meetings.
**End-of-life care** 37 points available (27 points for activity 10 points for network activity)	Demonstrate continuous QI activity focused on end-of-life care as specified in the QOF guidance and lead to improvements in the following aspects: Early identification and support for people with advanced progressive illness who might die within the next 12 months. Well-planned and coordinated care that is responsive to the patient’s changing needs with the aim of improving the experience of care. Identification and support for family/informal caregivers, both as part of the core care team around the patient and as individuals facing impending bereavement. Participate in a minimum of 2 network peer review meetings.

(From NHS England Guidance: NHS England, British Medical Association. 2019/20 General Medical Services (GMS) contract Quality and Outcomes Framework (QOF). Guidance for GMS contract 2019/20 in England)^12^

This study aimed to understand how practices were planning for and implementing the initial two QI modules. In this paper, we report findings of a rapid, qualitative study, aimed to capture the implementation phase at the start of the QI cycles in order to quickly support NHS England’s next steps on QOF QI development. We asked practice staff their perceptions of, and attitudes towards the QI modules, perceived barriers and enablers to implementation, what kind of change/resources/support would be needed to implement the modules and finally, if they thought QOF was a suitable vehicle for incentivising QI.

## Methods

We carried out a qualitative study using short, one-to-one semistructured interviews to gain insight into general practice attitudes to QOF QI modules and difficulties with, and support needed for, implementation. Practice managers were chosen as the source of data because they are more accessible for interview than clinical staff. The sample was found with the assistance of the database held by the RCGP Research and Surveillance Centre (RSC), a network of over 200 representative practices across England.^14^ The RSC sent an email to practices along with a participant information sheet, requesting expressions of interest in participating. Practices were offered a payment of £40 for taking part. Contact details of practice managers who had expressed interest were forwarded onto the research team. The research team then emailed an invitation to take part in a 20–30 min semistructured interview to all practices who had responded, aiming to recruit 30 practice managers.

### Semistructured interviews

Twenty-five semistructured telephone interviews were conducted with responding practice managers (July 2019 to November 2019). Interviews were conducted by two health services researchers, one based in the North of England (DB) and one in the South (SH) using the same interview guide. The interview guide was developed with the wider research team and designed to address the research questions (see online supplemental appendix 1 for interview schedule). Interviews were audio recorded (with consent) and then transcribed verbatim.

10.1136/bmjoq-2022-001960.supp1 Supplementary data

### Analysis

Analysis of the interviews was undertaken on an iterative and concurrent basis by the three team members, beginning as soon as data collection commenced and informed by post interview notes and transcribed recordings. This allowed for emerging findings to inform development of subsequent interview topic guides to probe themes deemed important. Anonymised, verbatim interview transcripts were imported into NVivo V.12 Plus, a qualitative data analysis software tool, to allow the research team to code the data to develop themes. Qualitative data obtained from the interviews were analysed using the framework method.^15^ This approach to thematic qualitative data analysis was specifically developed with applied policy research in mind, and in particular healthcare research, where the aims and objectives of the investigation are predefined, there are specific research questions to be answered and the projects are typically of limited duration requiring a rapid feedback of results. The framework approach enables this by being a systematic and structured way of managing textual data analysis whereby text is synthesised and data are ‘charted’ into a matrix in order to identify, define, interpret and ultimately explain, themes/concepts/associations across and between the qualitative data.^15^

In line with the framework approach, an initial index or analytical coding framework of a priori codes were developed informed by our knowledge of existing relevant research and our research questions. As thematic analysis proceeded and additional themes arose, the framework was augmented with additional codes. This ensured that the research team captured unexpected issues and phenomena. The framework was applied to the coding of each transcript and when viewed collectively, allowed for comparison between interviews in order to understand a range of factors relating to the implementation of the new modules. Coding was discussed at regular research team meetings to ensure consistency. Three main themes were identified and categorised as: awareness and early experiences of implementation; anticipated challenges to implementation and effects on staff; and the appropriateness of QI in the QOF.

### Patient and public involvement

Participants in the study were required to be familiar with the QOF which is specific to general practice in England. As such, patients and the public were not involved in the design, conduct, reporting or dissemination plans of our research.

## Results

We interviewed practice managers from 10 practices in London and the South, 7 in the Midlands and 8 in the North. Participants’ total length of employment with the NHS ranged from 7 months to 34 years with over half having 8 or more years’ experience. Their practices were larger and more likely to be rural than the English average in September 2019. The sample comprised practices with a mixed range of characteristics, for example, practice size, region, urban/rural, average socioeconomic status of patients, teaching and non-teaching as per table 2.

**Table 2:** Characteristics of practices taking part in phase 1 compared with English average^36^

		Practices taking part	All practices in England
		(n=25)	(n=6796)
Mean list size (patients)		10 716	8826
Range of list size (patients)		7961–44 303	1002–84 724
Rural (%)		40%	15%
Region	London and South	40%	40%
	Midlands	28%	30%
	North	32%	30%

Participants were willing to share their experiences on the introduction and implementation of the two new QI elements in QOF and on the changes to QOF more broadly. Participants’ involvement with QOF varied from leading the practice in this area to being involved on a limited basis via emails, meetings and providing monitoring of the system.

### Awareness and early experiences of implementation

All participants were aware of the incentivisation of QI in the QOF and all but one said they had started implementation. Many had already met with PCN colleagues about QI. Many reported delays in implementation because of receiving the business rules and guidance from NHS England late.

Feedback on the QI modules themselves was relatively positive, with many viewing the changes as good for patient care and in the case of prescribing safety, not onerous to implement because prescribing was a popular topic for QI activities already:

so for me personally I find that a really positive step, it links in with a lot of our local primary care contracts, you know with our CCGs, so we have a lot of prescribing…when you talk about prescribing safety, we have a lot of targets that we’re hitting outside of QOF anyway that they kind of link in with, so it’s quite useful, so if I think it’s positive… (PSH009)

However, the end-of-life care module was considered more challenging. Some participants mentioned being uncomfortable with surveying carers or patients at the end of life, which they felt may be insensitive:

Well the patient survey is probably the only thing that we're not particularly comfortable with and that’s because they're asking us to go back to families and start to unpick the palliative care and, one, they may not want to do it, and, two, they may actually have been perfectly happy but then you start to question it and you raise questions in their minds as to why you're asking it. (PDB001)

Some practice managers also felt it would be difficult to measure the impact of this module on quality of care, recognising that defining good end-of-life care and measuring improvement is challenging:

I think it will be difficult to measure that… There might be somethings like what referrals for the different services or like social prescribing or like, you know, talking to patients about their end of life and what they want to do and preparing the staff and sending training but again, how are you going to measure that from patients. (PDB013)

Participants thought that the shift away from metrics-based incentivisation was positive, and liked the fact that it was more than a tick-box exercise, more patient focused and more likely to bring standardisation across practices. One practice also mentioned that as the modules change annually, it will make practices less likely to be complacent although one participant thought the opposite by suggesting that:

I don’t know what change is going to be made by incentivising it for a year. What is that going to achieve? (PDB003)

### Anticipated challenges to implementation and effects on staff

Most practice managers felt only minor changes in organisation were needed to introduce the QI modules. However, some suggested that clinicians would need to put more time into the QOF because of QI, especially the end-of-life module:

…I don't think there is any practice anywhere that wouldn't want to improve the quality of care that they offer their patients. It’s a question of having the resource to be able to do that extra work. (PDB008)

Some participants suggested that working collaboratively with other practices might be difficult and it would take time to build relationships and trust across PCNs. However, working on initiatives across the PCN was seen as a positive opportunity to share best practice and promote consistency especially on end-of-life care across the country.

### The appropriateness of QI in the QOF

A few participants suggested that QI in the QOF simply enhanced existing activities in their practice but acknowledged that the module had forced them to evaluate current practice in a positive way. Concerns were expressed that a ‘one-size-fits-all’ approach to QI may not be optimal: while simple guidance is appropriate for some practices with limited experience, there is a risk of oversimplifying the requirements for practices that have been early adopters or have significant experience in QI:

….only criticism might be that every practice is very different and making us work as a network to do this work is perhaps not necessarily how we would have done it but I'm not saying it’s a good thing or a bad thing, but I hope it doesn’t dumb us all down. (PDB001)

Other participants suggested that the effort of QI in the QOF for clinicians may not pay off in terms of patient outcomes, especially in relation to the prescribing safety module, seeing medication reviews as standard practice anyway:

I think patients now are used to things like prescribing changes over the counter, so I don’t think they’ll think it’s anything different if they’re called in for a review based on something that we’ve done in a QI improvement module, it’s part and parcel of what we do now, especially having a clinical pharmacist. (PDB005)

Participants were particularly optimistic about the end-of-life module where they could envisage genuine benefits to reviewing current practice and implementing QI cycles:

I think the impact on the patient is only positive, it means that they’re getting a lot more focused care and if I take end of life as an example, it’s really focused our attention on how we are managing that indicator and what we are doing for our patients. So I only see it as a positive really for patient care. (PSH008)

Many appreciated the opportunity carry out activities that did not involve the ‘tick-box’:

I think that over the years, they’ve seen it as a tick-box exercise and so that is their main focus because that’s a lot of funding for practices. So if it is…so having it more patient-focused and personalised care plans is definitely better for the clinicians and for the patients, I think. (PDB006)

On the other hand, others pointed out the opportunity cost of focusing on one area of care potentially at the expense of others:

you know, you’re ticking the box to say, yes, we’ve delivered everything we’ve been asked to do but you could be spending a lot of time doing things that aren’t adding that much value where something else that isn’t incentivised so therefore doesn’t get done could have more. (PDB012)

A few participants questioned the choice of topics for the QI modules especially as many already undertake prescribing audits and quality initiatives:

I think there could be other areas that they could have perhaps picked up on more. More perhaps from the mental health side. (PDB003)

Additionally, there were those who expressed the view that there should be greater practice participation in the development of future QI modules. Others suggested that it may be appropriate for practices to have some flexibility to choose module topics and complexity of approach to ensure relevance to local need and to reflect existing QI skills and experience:

…specific to our needs and our patients. And maybe if nationally we’re not going well on something they should be in for everybody. But say in my area, nationally blood pressures are really bad in diabetic patients. (PDB004)

## Discussion

The new modules are an attempt to bridge the gap between a tick-box exercise and holistic patient care/health management as per the vision of the 2018 review of QOF.^4^ At the time of interview, most participants were implementing the QI modules in the QOF and had broadly favourable views of it, recognising that it had potential to improve the quality of care and welcoming the less ‘tick-box’ approach. There were some concerns about the extra clinical and managerial time needed and the challenges of working across PCNs. It should be noted here that PCNs were newly formed in 2019; a move that was not necessarily welcomed by general practice.^16^ It may be that introducing the QI modules in this context may have impacted on their implementation. The prescribing safety module was seen as relatively easy to implement because of practices’ previous experience with this topic; by the same token some felt that it should not be a priority for incentivised QI, because practices were doing it anyway. Several participants raised further issues about choice of topic, recognising the range of topics where QI may have greater potential for improving quality of care, and the need for local flexibility according to patient needs. Participants also highlighted that focusing on specific areas may mean that other areas of work (including other formal QI activities or other activities that might improve the quality of care) would be deprioritised.

### Comparison to existing literature

Pay-for-performance schemes similar to QOF that focus on achievement of defined quality standards are now commonly used in healthcare systems across the world.^17^ The evidence that financial incentivisation of achievement of quality standards improves health outcomes or quality of care (rather than simply more frequent delivery of incentivised activities) is weak, in the UK or elsewhere.^7 17–21^ There are few reports evaluating financial incentivisation of QI activities rather than incentivising achievement of standards. For example, in Scotland, QI is now implemented through the collaboration of practices in GP ‘quality clusters’ working on a locality rather than top-down basis. Their success has yet to be fully evaluated, but an initial report suggests that that their maturity is variable and that appropriate support is needed to conduct initiatives at the localised level.^22^ This has implications for our findings which suggest a preference for a localised approach to QI. A study in Sweden in which Stockholm general practices were financially incentivised to carry out QI projects, found that the number of QI projects reported more than doubled compared with the year before the intervention.^23^ It seems likely that this was due to the financial incentivisation, although in the absence of a control group it is not possible to attribute the outcome to the intervention with confidence. The effect of increasing QI activity on patient outcomes was not reported.

The evidence that QI in primary care improves clinical processes or patient outcomes is mixed, although the evidence suggests that it may be marginally more effective in primary than secondary care.^24 25^ There are calls for the increased robustness of research about QI, as well as more systematic drawing of lessons from evaluation of QI practice.^25^ Specific elements of QI are considered effective, in particular audit and feedback.^26^ Other practice-level interventions to promote high quality care, including educational outreach and continuing education meetings, have also been found to be modestly effective at improving relatively easily measurable process and health outcomes.^27^ There is a growing evidence base suggesting that local incentivisation schemes may be effective,^28^ and may be better able to address local needs and gaps in quality of care.^29^ A sense of local ownership may be more likely to motivate behaviour change.^30^ Analysts of policy on this issue argue that ‘a key approach to QI is fostering, perhaps even demanding, local responsibility for QI, but not imposing the precise approach and measures that the local actors have to use’.^31^ Our findings also support evidence which suggests that context is important for QI programmes;^25 32^ financially incentivised QI in England may need to better adapt to organisational context or local needs.

What our study has highlighted, interventions in primary care aiming to improve quality of care tend to focus on specific projects, specific disease outcomes or specific clinical processes, not taking a broader view of what constitutes high-quality primary care. Defining high quality primary care, as has been attempted by a Canadian consensus exercise^33^ is challenging, but must take into account broader issues, for example, continuity of care, patient empowerment or support for self-management (not necessarily for specific diseases).

### Strengths and limitations

While data collection was timely, enabling us to capture early learning about challenges and enablers, the sample size was small. Practice managers cited heavy workloads and workload associated with the influenza vaccination campaign as preventing them from taking part and therefore meeting the sample threshold of 30 was a challenge. Practices were self-selecting, and other practices may not be as QI-ready as those involved in our research and it is possible that working with colleagues in other practices in the PCN is less developed elsewhere. The findings may, therefore, not be generalisable to all practices and others may find the QI modules more challenging to implement. Our study was a preliminary investigation with limited findings and we are aware that we have not included the clinician perspective at this stage. Practice managers were considered to be more accessible for a short term project such as this than clinical staff. The COVID-19 pandemic curtailed follow-on data collection with GPs to capture their views; this will be included in the subsequent phase of our study.

### Implications for research and practice

Financially incentivising QI interventions in the QOF may mean that practices are under pressure to implement them, in order to maximise practice income. This is likely to lead to deprioritisation of non-incentivised activities, as occurs with QOF.^34^ To tackle these issues, it may be appropriate to consider more locally flexible approaches to incentivisation of QI and other ways of improving quality of care. Focusing on the principles rather than the practice of QI may be more effective—for example, leadership and organisational commitment. Dixon-Woods and Martin suggest that QI should focus on ‘building capacity for designing and testing solutions’, and consider ‘programmes of activity and resources’ rather than projects.^35^ One way of building capacity and building in local flexibility might be to appoint PCN-level QI officers who focus on organisational commitment and capacity-building, support projects while strategically considering programmes of work, consider more broadly what constitutes high quality care and how to achieve it, and maintain a local focus.

### Conclusion

In summary, practices supported the idea of financial incentivisation of QI, however, it will be important to ensure that focus on QI cycles in specific clinical areas does not have unintended effects. Sustaining momentum with the introduction of new modules each year which are time consuming to carry out for time poor GPs/practices, will be a key issue going forward.

We plan next to evaluate the inclusion of the QI modules in the QOF over a longer period of time to February 2023 following the suspensions to QI in the QOF as a result of the COVID-19 pandemic.

## Data Availability

Data are available on reasonable request.
