# Supplementary material for: Evaluation of the introduction of QOF quality improvement modules in English general practice: early findings from a rapid, qualitative exploration of implementation
Source: BMJ Open Qual. 2022 Sep 26;11(3):e001960. doi: 10.1136/bmjoq-2022-001960 (PMC9516148; doi:10.1136/bmjoq-2022-001960)
Supplement: Supplementary data [file bmjoq-2022-001960supp001.pdf]

QOFQI Phase 1
Interview Schedule - Practice Managers

Role/context:

1 How long have you been a Practice Manager?
2 What is your involvement in QOF at your practice?

General View

3 Are you aware of the changes to the QOF? Are you aware of the introduction of the 2 new Quality Improvement modules and Personalised care adjust that replaced exception reporting April 2019?
4 What about other staff in the practice? Do they know about it?
5 What are your thoughts on the changes?
6 What about other staff in the practice?

QI Modules Specific

7 What are the plans of the practice to implement the QI modules?
8 - How are the plans going?
9 - What kinds of changes will be needed to accommodate QI activities in the practice?
10 - Do you think the practice has sufficient resources to implement and administer QI activities?
11 - What kind of support do you need to implement the QI modules?
12 - Likewise, do practice staff feel supported to implement the QI modules?
13 Do you have any concerns about implementing the modules?
14 - Can you envisage any factors that might particularly hinder you?
15 - Are there any factors that might be helpful to implementation?

QOF General

16 How do you/staff feel about the shift away from the standard metrics-based QOF indicators?
17 Do you/practice staff think that QOF is a suitable method for incentivising QI?
18 - If not why not?
19 - Do you have any thoughts on an alternative approach or system of incentivisation?

Outcomes

20 What do you think the impact of the changes will be for:

- patients and on patient care?

- staff?

21 Are there any other issues about the QOF changes you would like to tell me about that we haven't covered today?

22 Finally, is there anyone else in the practice we should be talking to about this, if so, please could we have their details?

Thank you for talking to me, it's very much appreciated!

Please could you tell us if you would mind us contacting you again for further information? No obligation.
